# A young lady presenting with acute limb ischemia secondary to systemic lupus erythematosus, antiphospholipid syndrome and infective endocarditis

**DOI:** 10.12669/pjms.40.3.8736

**Published:** 2024

**Authors:** Alia Ali Muhammad, Javeria Shah

**Affiliations:** 1 Dr. Alia Ali Muhammad, MBBS, FCPS (Med) Assistant Professor, Shaikh Zayed Postgraduate Institute, Lahore, Pakistan; 2 Dr. Javeria Shah, MBBS. Postgraduate Trainee, Medicine, Shaikh Zayed Hospital, Lahore, Pakistan

**Keywords:** Acute Limb Ischemia (ALI), Systemic Lupus Erythematosus (SLE), Antiphospholipid syndrome (APLS), Infective Endocarditis (IE)

## Abstract

We report a case of a 35 years old lady presenting with acute upper limb ischemia secondary to systemic lupus erythematosus (SLE), antiphospholipid syndrome (APLS) and infective endocarditis (IE). It is rare for SLE/APLS to present with acute limb ischemia (ALI) as the initial manifestation. The patient presented with high grade fever along with pain and numbness in her right upper limb. On examination her right upper limb was cold to touch and the peripheral pulses were not palpable. There was also an audible pansystolic murmur in the mitral area. CT Angiography confirmed a complete occlusion of the right axillary artery while echocardiogram revealed severe mitral regurgitation with large vegetations on the mitral valve leaflets, suggesting infective endocarditis.

After the patient’s clinical deterioration and considering the severity of the ischemic condition, additional investigations were conducted, which ultimately led to the diagnosis of SLE with APLS. Management included antibiotic therapy for IE and high dose of IV steroids and anticoagulants for SLE/APLS, to which she responded well. This case emphasizes the significance of conducting a comprehensive evaluation of all possible causes of acute limb ischemia, while considering the patient’s medical history and physical examination findings.

## INTRODUCTION

Systemic lupus erythematosus (SLE) is a systemic autoimmune disease characterized by the production of antinuclear antibodies and multisystem involvement, leading to significant morbidity and mortality.^1^

Antiphospholipid antibodies can be detected in up to 40% of SLE patients, although only half of them will experience thrombosis and/or miscarriages.^2^ Antiphospholipid syndrome (APLS) is a disorder that manifests as venous or arterial thrombosis, pregnancy loss, and the presence of antiphospholipid antibodies. The lower limbs and the cerebral arterial circulation are the most common sites of venous and arterial thrombosis observed in APLS, respectively.^3^ It is worth noting that arterial thrombosis is less frequent than venous thrombosis in APLS.^4^

This report describes a challenging case of upper limb arterial thrombosis in a patient with SLE, APLS, and infective endocarditis. The complexity of diagnosing this case shows the need for effective diagnostic and management strategies. It also emphasizes the importance of considering SLE, APLS and infective endocarditis as potential underlying causes of arterial thrombosis in the upper limb. This report provides a detailed analysis of the case and outlines possible diagnostic and treatment approaches that will aid in managing similar cases in the future.

## CASE REPORT

A 35 years old lady presented with a four weeks history of high-grade fever and 10 days of pain and numbness in the right upper limb. The patient reported being well one month prior but experienced a spontaneous abortion during the second trimester, after which she began to feel unwell and developed a high-grade fever. Subsequently, she experienced numbness and severe pain in her right arm. She reported a history of another spontaneous abortion two years prior during the first trimester of pregnancy. The patient had no other medical complaints and denied any autoimmune symptoms such as oral ulcers, photosensitivity, or Raynaud’s phenomenon. She was not taking any oral contraceptives.

Upon presentation in the emergency room, the patient appeared pale and emaciated, and was febrile with a temperature of 101°F, a pulse of 120/min, and a blood pressure of 110/70. The patient’s right arm was cold to the touch with absent radial and brachial pulses, and an area of slight bluish discoloration was observed at the tip of the right index finger. Sensory and motor examinations of the arm were normal. Notably, an audible pansystolic murmur was detected in the mitral area, which radiated to the axilla.

Investigations revealed a hemoglobin level of 7.9 g/dL (normocytic, normochromic), a total leukocyte count of 18 x 10^9/L, and a platelet count of 88 x 10^9/L. Urine examination showed the presence of protein with a 24-hour urine protein level of 39 mg, and the presence of blood**++**. The patient’s liver function tests, renal function tests, and serum electrolytes were all within normal limits. An echocardiogram showed severe mitral regurgitation with mitral valve prolapse, along with highly mobile, large vegetations on the anterior mitral valve leaflet, suggestive of infective endocarditis. Blood cultures were negative, which was attributed to the administration of empiric antibiotic therapy.

**Fig.1:**
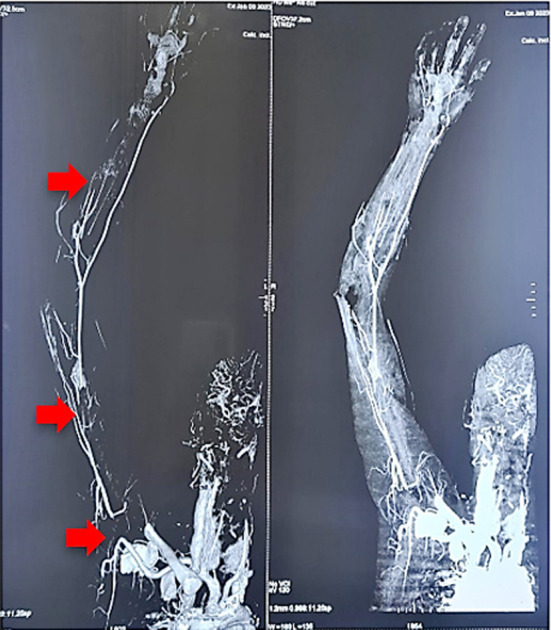
CT angiogram, showing thrombosed axillary, brachial and ulnar arteries.

**Fig.2:**
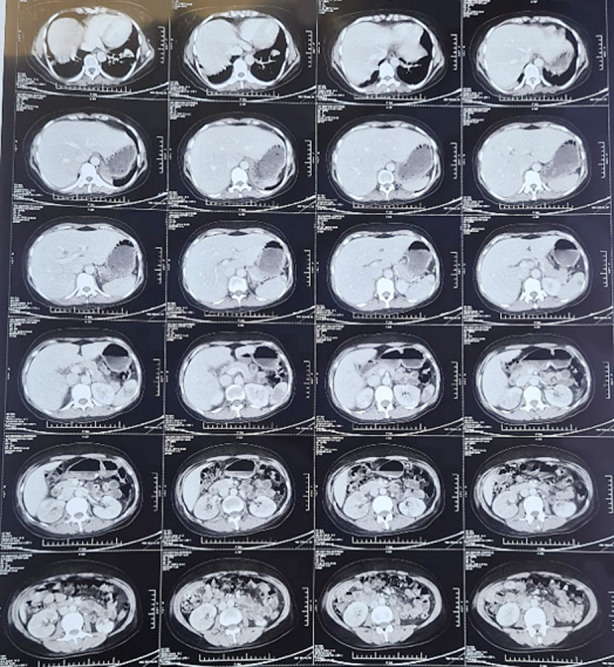
CT abdomen showing splenic and renal cortical infarcts.

A CT angiogram was performed to investigate the absent pulses in the patient’s limb. It revealed thrombosis-related complete occlusion of the axillary artery, the distal brachial and ulnar arteries, with the formation of collaterals (Fig.1, indicated by red arrows). CT abdomen was also undertaken which showed multiple splenic infarcts and few cortical infarcts in kidneys bilaterally (Fig. 2).

We suspected acute limb ischemia (possibly secondary to SLE/APLS related thrombosis or infective endocarditis related septic emboli) and started anticoagulation therapy with warfarin bridged with heparin. Empiric intravenous antibiotic treatment with ceftriaxone, vancomycin, and low-dose gentamicin was initiated, and blood cultures were obtained. Repeat echocardiogram showed reduced vegetation size after two weeks and complete resolution after four weeks of antibiotic therapy. However, her clinical condition did not improve much and her fever persisted. Given her history of two previous abortions and severity of the ischemic condition, we ordered an ANA test, which returned strongly positive with a titer of 1:1280. C3 levels were normal, while C4 levels were low. Serum Anti-ds DNA was positive with a value of 48.6IU/ml (a positive result is equal to or above 25), and lupus anticoagulant antibodies were moderately positive (62.9 seconds), with positive anticardiolipin IgG. The patient received pulse therapy using IV Methylprednisolone, with a total dosage of 3g. Following treatment, the patient’s fever subsided, and she began to feel better. After completing pulse therapy, the patient was discharged and continued on oral warfarin to maintain an INR level between 2-3. She was referred to a cardiothoracic surgeon for management of her MR and to a rheumatologist for the management of her underlying autoimmune disease.

During follow-up, it was observed that the tip of the patient’s right index finger was dry and gangrenous, with a clear demarcation mark, and there was a possibility of auto-amputation, her peripheries were otherwise warm to touch and INR was 2.8. Mitral valve replacement was planned as part of the patient’s ongoing treatment plan for severe mitral regurgitation.

### Consent:

All images included were taken and added after getting patient’s consent.

## DISCUSSION

Patients with severe infective endocarditis are at risk of major arterial emboli, which can affect any part of the body, but certain organs and regions such as the liver, spleen, brain, and lower extremities are more commonly affected. Septic emboli can occur at any time during IE and may be the first presenting sign.

Valvular disease is prevalent in SLE and is characterized by Libman-Sacks vegetations that cause valvular abnormalities. This increases the risk of infective endocarditis and thromboembolic events. However, the role of antiphospholipid antibodies in SLE-associated valvular lesions remains inconsistent in the literature.^6^

Antiphospholipid Syndrome is a systemic autoimmune disease that can cause both thrombotic and non-thrombotic symptoms. Although it is rare, APLS can sometimes present as acute limb ischemia.^7^ Previous case reports have shown a link between arterial thrombosis in the lower limbs and both SLE and APLS.^8^,^9^ However, upper limb thrombosis is a rare occurrence and only a few case reports have been published.^10^

This case report emphasizes the importance of considering limb ischemia as a possible presentation of SLE with APLS, especially when accompanied by infective endocarditis or other associated conditions. Early diagnosis and prompt management, involving a multidisciplinary approach with rheumatologists, cardiologists, and vascular surgeons, can help prevent further complications and improve the patient’s prognosis.
